# Psychometric Properties and Criterion Validity of STEU-B and STEM-B in Chinese Context

**DOI:** 10.3389/fpsyg.2019.01156

**Published:** 2019-06-06

**Authors:** Shuqun Yan, Yuting Feng, Yaoshan Xu, Yongjuan Li

**Affiliations:** ^1^CAS Key Laboratory of Behavioral Science, Institute of Psychology, Chinese Academy of Sciences, Beijing, China; ^2^Department of Psychology, University of Chinese Academy of Sciences, Beijing, China

**Keywords:** emotional understanding, emotional management, situational judgment test, item response theory, criterion validity

## Abstract

Emotional intelligence (EI) has attracted increasing attention in organizational psychology. The aim of this study was to test the applicability of two performance-based emotional intelligence tests developed in western countries, namely, the brief versions of the Situational Test of Emotional Understanding (STEU-B) and the Situational Test of Emotional Management (STEM-B), in a sample of 904 Chinese employees. Specifically, item response theory (IRT) analyses were conducted. The item parameters along with the item and test information functions of the Chinese versions of the STEU-B and STEM-B were estimated. Moreover, the associations between the STEU-B and STEM-B scores and several work-related variables were examined. The results showed that the STEU-B and STEM-B had acceptable internal consistencies, and similar mean proportions of correct responses, item parameters, item information functions, and test information functions in China, as reported in previous studies. Furthermore, the scores were found to be related to the employees’ psychological strain, job-related affect, job satisfaction, and supervisor-rated job performance in a theoretically hypothesized manner. These findings suggested that the STEU-B and STEM-B might be useful measurements in future EI studies in the Chinese organizational context.

## Introduction

There is a growing interest in emotional intelligence (EI) in social and organizational psychology, and an increasing number of empirical studies have focused on the criterion validity of EI in predicting real-life outcomes. The EI label has been historically applied to two relatively distinct theoretical constructs: ability EI and trait EI. Ability EI refers to “the ability to perceive emotions, to access and generate emotions so as to assist thought, to understand emotions and emotional knowledge, and to reflectively regulate emotions so as to promote emotional and intellectual growth,” which emphasizes EI as an actual ability (Mayer and Salovey, 1997). Trait EI refers to self-perceived emotionality and emotional efficacy that is located within the personality domain (Kafetsios and Zampetakis, 2008). There is evidence of the criterion validity of both ability and trait EI. Ability and trait EI have been found to play important roles in stress management and adaptive coping (Ciarrochi et al., 2002; Oginska-Bulik, 2005), interpersonal relationships and social networks (Brackett et al., 2006; Gallagher and Vella-Brodrick, 2008), intimate relationships (Brackett et al., 2005), and academic achievement (Van Rooy and Viswesvaran, 2004). In the workplace, employees with a high degree of trait EI have been shown to experience more positive and less negative affect (Kafetsios and Zampetakis, 2008), to be more satisfied with their jobs (Kafetsios and Zampetakis, 2008; Greenidge et al., 2014; Meisler, 2014), and to exhibit better job performance (Greenidge et al., 2014; Mulki et al., 2015). A meta-analysis also found that ability EI and trait EI were positively correlated with job performance (O’Boyle et al., 2011). Moreover, empirical evidence revealed that both ability EI and trait EI could act as buffers between job stressors and psychological health (Ciarrochi et al., 2002).

In line with the above definitions, the measurements methods of the two forms of EI are different. Ability EI is assessed through performance-based measurements resembling standard intelligence tests, in which respondents are instructed to maximize effort to achieve the maximum performance on problems related to emotional abilities (Côté, 2014). Trait EI is measured by self-report instruments, through which respondents are asked to confidentially evaluate the contents that describe their abilities in the emotional domain (Schutte et al., 1998). The accuracy of the responses to the self-reported EI items depends on whether the respondents are able to accurately estimate their abilities related to emotional processes and whether they are willing to report them (Côté, 2014). However, evidence has shown that individuals may overestimate their EI (Brackett et al., 2006; Sheldon et al., 2014). Moreover, the self-reported EI questionnaires are susceptible to social desirability bias. For example, applicants may fake their trait EI in these questionnaires during personnel selection. Therefore, EI researchers encourage the use of performance-based measurements to capture actual EI abilities in research and practice, especially in organizational settings (Côté, 2014). Thus, the current study mainly focused on ability EI.

The most prevalent theoretical model in the ability EI research domain is the hierarchical four-branch model, which proposes four branches of ability EI: perceiving/expressing emotions (i.e., accurate perception and expression of emotions); using emotions (i.e., capitalizing on the systematic effects of emotions on cognitive activities); understanding emotions (i.e., identifying the connections between emotions and events); and regulating emotions (i.e., increasing, maintaining, or decreasing one’s own or others’ emotions) (Mayer and Salovey, 1997). Based on this model, Mayer et al. (2002) developed the Mayer-Salovey-Caruso Emotional Intelligence Test (MSCEIT) to measure these four EI branches. To date, research on ability EI has been dominated by the MSCEIT, and thus, what we know about ability EI is largely based on this measurement. However, it is difficult to know whether these empirical results were attributable to the constructs examined or the unique measurement method used. Moreover, there is evidence to suggest that the MSCEIT has problems with its scoring method (Austin et al., 2008), as well as with its task and item selection (Roberts et al., 2006), which emphasizes the necessity and importance of developing alternative measures of ability EI.

To provide alternative instruments for assessing ability EI, MacCann and Roberts (2008) developed the situational test of emotional understanding (STEU) and the situational test of emotional management (STEM) using the situational judgment test paradigm. The STEU and STEM target the third and the fourth branch of the four-branch ability EI model, respectively. According to this model, the four hierarchically ordered EI branches monotonically increase in cognitive complexity from the first to fourth branch, and can be grouped into two areas: experiential EI (encompassing the lower two branches) and strategic EI (encompassing the two higher branches) (Mayer et al., 2002). Thus, the STEU and STEM provide a comprehensive picture of strategic EI. The understanding emotions branch is the “most cognitively saturated” and regarded as the key focus of abstract processing and reasoning with respect to emotion (Mayer et al., 2001). The regulating emotions branch is the highest and most complex branch; it involves managing emotions for personal and interpersonal growth, which combines and balances motivational, emotional, and cognitive factors (Mayer et al., 2001). A recent empirical study indicated that the discriminating and predictive power of ability EI lay primarily in these two strategic branches (Dimitrijević et al., 2018).

The STEU measures an individuals’ ability to understand the connections between events and emotions (i.e., the understanding emotions branch) (MacCann and Roberts, 2008). The content of the items of the STEU was derived from Roseman (2001) appraisal theory, which provided a strong theoretical basis for emotional understanding. Within the framework of this theory, individuals’ evaluation of a situation or event cause specific reactions and bring about emotional responses based on their appraisal, and 17 discrete emotions are generated according to specific combinations of seven appraisal dimensions (motive-consistency, causal attribution, certainty, control potential, unexpectedness, motivational state, and problem source). The STEU consists of 42 scenarios covering the following emotions: sadness, pride, relief, joy, regret, gratitude, distress, hope, contempt, surprise, frustration, anger, fear, and dislike. The scenarios contain ample multiple-choice items, including 14 context-reduced items, 14 with a personal-life context, and 14 with a workplace context (MacCann and Roberts, 2008). In each scenario, an emotional situation is described, and five emotions are presented. Respondents are asked to indicate which emotion is most likely to be generated by that particular situation. The answers of the items are scored as either correct or incorrect based on the appraisal theory. Thus, the scoring system of STEU is theoretically based and substantially different from the scoring system used for the MSCEIT. The STEM measures individuals’ ability to cope with stressful events by regulating negative emotions and enhancing positive emotions through emotional management (i.e., the regulating emotions branch), which is developed on the basis of the situational judgment test paradigm. In accordance with this paradigm, items were generated by the semi-structured interviews, and answers from participants about those items constituted the response options. The relevant experts decided the scoring system based on their selection for the proportion of each option (MacCann and Roberts, 2008). The test consists of 44 scenarios covering three emotions, namely, fear, anger, and sadness. In each scenario, an emotional situation is described and four options regarding the action to manage the emotions and solve the problems in that scenario are presented. The respondents are asked to select the most effective option. The STEU and STEM showed good convergent and divergent validity. The correlation between the STEU and STEM scores was 0.29 (Austin, 2010). The STEU scores correlated at 0.44 with the MSCEIT understanding scores (Austin, 2010) and at 0.31 with scores on the theory of mind test (Ferguson and Austin, 2010). The STEM scores correlated at 0.30 with the MSCEIT management scores (Austin, 2010) and at 0.21 with scores on the theory of mind test (Ferguson and Austin, 2010). The STEU and STEM also showed small to moderate correlations with personality traits (MacCann and Roberts, 2008; Libbrecht and Lievens, 2012). Moreover, the STEM scores correlated at 0.23 with academic performance in a sample of undergraduate medical students (Libbrecht et al., 2014), thus providing support for criterion validity in real life.

More recently, researchers have developed the brief version of STEU (STEU-B) and the brief version of STEM (STEM-B) by evaluating the psychometric properties of STEU and STEM using the item response theory (IRT) method (Allen et al., 2014, 2015). IRT provides valuable methods for assessing the psychometric properties of EI measurements (Karim, 2010; Cho et al., 2015), which has advantages compared with the classical test theory (CTT) method. First, unlike CTT, which examines the psychometric properties of EI measurements based on observed scores, the IRT method provides psychometric information that is not dependent on the sample. Furthermore, the CTT method assumes a constant effectiveness and measurement precision of the test and items. In comparison, the IRT method holds that the effectiveness and precision of the test and items vary across different levels of the trait. Therefore, the IRT can be used to calculate the probability that the respondents choose a particular answer of each item and to estimate the ability of the test and each item to differentiate respondents at every level of EI. Allen and colleagues evaluated the item parameters (i.e., discrimination, difficulty, and guessing parameters) and the item information for each item included in STEU and STEM as provided by IRT analysis (Allen et al., 2014, 2015). Based on these psychometric properties, the items with low “maximum effectiveness” (a maximum amount of item information < 0.05) and providing information for similar areas of the latent scale were omitted, resulting in 19-item STEU and 18-item STEM scales. Thus, the STEU-B and STEM-B can provide sufficient information across different levels of item difficulty. The Cronbach’s alpha coefficients for STEU-B and STEM-B were 0.63 (Allen et al., 2014) and 0.84 (Allen et al., 2015), respectively. The correlation between STEU-B and STEM-B was 0.30 (Allen et al., 2015). With the increasingly high usage of EI measurements in research and practice, the short version of performance-based EI instruments has been requested by both EI researchers and organizational managements. Thus, STEU-B and STEM-B can be useful tools in cases where research time is limited and for organizational management purposes.

Despite these significant advances in EI research, STEU and STEM research has been limited to Western cultural participants (e.g., MacCann and Roberts, 2008; Austin, 2010; Côté et al., 2011). Previous evidence has indicated that cultural differences between performance-based EI tests may exist (Côté, 2014). Therefore, the generalization of STEU-B and STEM-B should be further examined in different cultural contexts. Furthermore, EI is an increasingly important issue in the workplace setting, and applying EI instruments in organizational management comes with the growing need to evaluate the measurement precision and criterion validity of EI instruments in the organizational setting (Karim, 2010; Greenidge et al., 2014). However, empirical evidence for the criterion validity of STEU-B and STEM-B to predict the work-related variables in a real organizational setting is limited. It is also unknown whether the patterns of associations between EI and work criteria that have been found in research on Western culture hold in Chinese organizational context. Accordingly, this study aimed to validate the STEU-B and STEM-B in a sample of Chinese employees in terms of psychometric properties and criterion validity. Specifically, we analyzed the psychometric properties of the Chinese versions of STEU-B and STEM-B using the IRT method and examined the associations between the Chinese versions of STEU-B and STEM-B scores and several work-related variables. By doing so, this study improved the research on EI in different cultural contexts and extended the information on the STEU-B and STEM-B by providing their criterion-related validity in the Chinese organizational setting.

Specifically, we expected the Chinese versions of the STEU-B and STEM-B scores to be related to the work-related criterion in several respects. First, we posited that EI scores should be negatively associated with the indicators of occupational stress and strain. The abilities of emotional understanding and emotional regulation facilitate stress management and adaptive coping (Oginska-Bulik, 2005; Gallagher and Vella-Brodrick, 2008). Thus, employees who are capable of understanding and regulating emotions can cope with negative events and occupational stress well, and thereby suffer less psychological strain than employees with low EI levels. Second, EI should be related to positive and negative affect at work. To be specific, the emotional regulation branch of EI can help employees to cope with high job demands and undesirable job-related events, as well as to control and alter emotional experiences caused by unfavorable events, which may lead to more positive experiences and less negative experiences at work. Consistent with this reasoning, evidence has shown that employees with high ability of emotional regulation experienced more work-related positive affect and less work-related negative affect than employees with low emotional regulation ability (Kafetsios and Zampetakis, 2008; Parke et al., 2015). Third, we posited that emotionally intelligent employees should be more satisfied with their jobs. Employees with high abilities of emotional understanding and regulation can better understand and anticipate others’ emotions in the workplace, cope with negative experiences and unfavorable job-related events, and have better psychological health than others (Sy et al., 2006; Vratskikh et al., 2016). This can in turn increase their job satisfaction levels. Existing research has consistently suggested that EI predicts employees’ job satisfaction (Kafetsios and Zampetakis, 2008; Greenidge et al., 2014; Ouyang et al., 2015; Vratskikh et al., 2016). Thus, the STEU-B and STEM-B scores should be positively associated with employees’ job satisfaction. Fourth, EI is an important predictor of job performance. In two meta-analyses, the correlations between performance-based EI scores and job performance were 0.16 (Joseph and Newman, 2010) and 0.21 (O’Boyle et al., 2011), respectively. Moreover, emotional understanding and emotional regulation were found to play different roles in determining job performance. In the cascading model of EI (Joseph and Newman, 2010; Newman et al., 2010), emotional understanding was proposed as an effect on emotional regulation, which in turn influenced job performance directly. Therefore, emotional regulation mediated the effect of emotional understanding on job performance. Accordingly, STEU-B and STEM-B scores should be positively related to job performance. Moreover, the association between STEM-B score and job performance should be stronger, and the effect of STEU-B score on job performance would be fully mediated by the STEM-B score.

In summary, based on the existing results of STEU and STEM, and the research on EI in the organizational context, the following hypotheses were proposed: (1) A significant correlation exists between the Chinese versions of STEU-B and STEM-B scores; (2) The Chinese versions of the STEU-B and STEM-B scores are negatively correlated with psychological strain; (3) The Chinese versions of the STEM-B score are positively correlated with positive affect at work and negatively correlated with negative affect at work; (4) The Chinese versions of the STEU-B and STEM-B scores are positively correlated with job satisfaction; and (5) The Chinese versions of the STEU-B and STEM-B scores are positively correlated with job performance, and the effect of the STEU-B score on job performance is fully mediated by the STEM-B score.

## Materials and Methods

### Participants and Procedures

The sample for this research was drawn from full-time employees working in an information technology company located in three major cities (Beijing, Shanghai, and Guangzhou) of China. Before the study, we contacted the company’s human resource management department to help us to distribute the survey. The employees were invited to participate in the study voluntarily. Participants went to a meeting room during their break time and were briefed on the purpose and procedure of the current study by a researcher individually. They were also assured that their responses would be kept anonymous and confidential. Then each participant provided written, informed consent prior to data collection. After that, they were asked to complete the STEU-B, STEM-B, and to participate in the measurement of criterion-related variables individually in the meeting room. In total, 904 participants completed and returned the survey. The sample consisted of 537 men and 367 women with an average age of 27.72 years (*SD* = 3.30) and an average job tenure in the current company of 4.20 years (*SD* = 2.52). The education level of the sample was relatively high; 25 participants (3.2%) had a high school diploma, 648 participants (82.4%) had a college education, and 113 participants (14.4%) had a master’s degree. The employees were from various departments: marketing and sales (20.0%), technology and data analysis (22.5%), product development (12.4%), customer service and consulting (13.8%), administration (12.5%), human resources (3.5%), finances (6.7%), and unspecified other departments (8.6%). Among these employees, 378 (41.8%) needed to interact with customers (e.g., sales, customer service technicians, and product managers), 438 (48.5%) required frequent team discussion and cooperation (e.g., consultors, product managers, and products technicians), and 85 (9.4%) were team leaders. The direct supervisors of the participants were invited to confidentially evaluate the job performance of their subordinates. We received 632 supervisor evaluations.

All of the procedures performed in studies involving human participants were approved by The Ethics Committee of the Institute of Psychology of the Chinese Academy of Sciences. Approval of the study was also done by the human resource management department of the company at which this study was conducted.

### Measures

The STEU-B and the STEM-B are described in the Introduction section. The English versions of the STEU-B and STEM-B were translated and adapted to the Chinese language in several stages. First, the original English versions were translated into Chinese by three bilingual native Chinese researchers independently. This resulted in different initial versions, which were reviewed and compared to produce consensual versions of STEU-B and STEM-B by the authors of the present study. Then, another bilingual native Chinese researcher back-translated these Chinese versions into English. The backward translator was familiar with the Chinese and western cultures and had no access to the original English versions. Next, the back-translated English versions were compared with the original English versions. Items with problematic back translations were thoroughly discussed by the authors and other experts in the field of emotion through a series of group meetings, and some minor revision were made to ensure the culture equivalence between the original English versions and the Chinese versions. Most modifications were minor, involving the choice between two synonyms or the change of the word order. The STEU-B was scored according to the original scoring system. Specifically, the correct answer was scored as “1” and the other answers were scored as “0” (MacCann and Roberts, 2008). The STEM-B scoring system is ordinarily based on the experts’ proportion of choosing each answer (MacCann and Roberts, 2008). In this study, we used the dichotomous scoring suggested by Allen et al. (2015) so that the IRT analyses could be conducted. Specifically, the best option was scored as “1,” and the other answers were scored as “0.”

The Chinese version of the General Health Questionnaire (GHQ-12) (Wang and Lin, 2011) was used to measure the psychological strain of employees. The questionnaire consisted of 12 items. Participants evaluated the levels of their psychological strain on a 7-point Likert scale (from 1 = strongly disagree to 7 = strongly agree), with higher scores indicating a higher level of psychological strain.

The IWP Multi-Affect Indicator (Warr et al., 2014) revised by Li et al. (2017) in the Chinese organizational context was used to assess participants’ experience of work-related positive and negative affect. This scale defined affect at work into four states: high-activation pleasant affect (HAPA), low-activation pleasant affect (LAPA), high-activation unpleasant affect (HAUA), and low-activation unpleasant affect (LAUA). Each dimension was measured using four adjectives that described work-related affect (HAPA: being enthusiastic, excited, inspired, and joyful; LAPA: being at ease, calm, laid back, and relaxed; HAUA: being anxious, nervous, tense, and worried; and LAUA: being dejected, depressed, despondent, and hopeless). The participants rated their experience at work in the past 4 weeks on a 7-point Likert scale (from 0 = never to 6 = always). As recommended by Warr and Parker (2010), the four single-quadrant scores were combined to create four double-quadrant dimensions: the positive affect dimension (all pleasant affect items) with higher scores indicating a higher level of positive affect, the negative affect dimension (all unpleasant affect items) with higher scores indicating a higher level of negative affect, the anxiety-comfort dimension (LAPA and reverse-scored of HAUA) with higher scores indicating a higher level of comfort, and the depression-enthusiasm dimension (HAPA and reverse-scored of LAUA) with higher scores indicating higher level of enthusiasm.

The job satisfaction scale developed by Schriesheim and Tsui (1980) was also employed. The scale consisted of 6 items. Respondents indicated their satisfaction with different aspects of their current job (e.g., co-workers, supervisors, and promotion) on a 5-point Likert scale (from 1 = very unsatisfied to 5 = very satisfied).

Supervisors were then asked to evaluate the general job performance of their subordinate on a 4-point scale (1 = fails, 2 = needs improvement, 3 = succeeds/meets standards, 4 = excels/exceeds standards). This measurement originated from Leavitt et al. (2011).

Demographic data on the employees (i.e., gender, age, and job tenure) were collected as control variables.

### Data Analysis Procedure

Descriptive statistics (mean scores and standard deviation), item-total score correlation indexes, and Cronbach’s alpha coefficients were computed.

Before the IRT analysis, the unidimensionality of the scale had to be examined because IRT assumes that the items included in the scale assess a single construct. Therefore, confirmatory factor analyses (CFA) were conducted to verify the unidimensionality of the STEU-B and STEM-B data.

IRT analyses were then conducted using the latent trait modeling package of R software (Rizopoulos, 2006). According to the dichotomous nature of the data, the 3-parameter logistic (3-PL) IRT model (Birnbaum, 1968) was used to fit the STEU-B and STEM-B items. With the 3-PL IRT model, the discrimination, difficulty, and guessing parameters were calculated. The discrimination parameters (ai) captured the relationship between the probability of endorsing the correct option for each item and the latent construct, which represented the discriminating power of the particular item. The discrimination parameters were interpreted qualitatively with the Baker (1985) classification using the following terms: *a* < 0.20, very low discrimination; 0.21 < *a* < 0.40, low discrimination; 0.41 < *a* < 0.80, moderate discrimination; 0.81 < *a* < 1, high discrimination; *a* > 1, very high discrimination. The difficulty parameters (bi) indicated the 𝜃 value (i.e., the latent trait) at which people had a 50% chance of selecting the correct answer and at which point the item could provide sufficient information. The guessing parameters (ci) represented the index of correct guessing, which reflected the probability of choosing the correct answer.

The item information curve (IIC) for each item was generated based on the IRT parameters, which described the distribution of information provided by an item across the continuum of the latent trait (𝜃). The area under IIC equaled the amount of information that the particular item could provide across the different levels of the latent trait. The amount of information indicated the ability of the item to distinguish the respondents with different levels of EI. The test information function (TIF) of the scale was calculated by aggregating the IICs of all items within the scale. The area under TIF represented the total test information.

To investigate the criterion-related validity of the Chinese versions of STEU-B and STEM-B, the partial correlations between the STEU-B score, STEM-B score, as along with the psychological strain, job-related effects, job satisfaction, and general job performance by controlling gender, age, and job tenure were calculated. Moreover, since the different effects of the STEU-B and STEM-B scores on job performance were expected, we conducted a hierarchical regression analysis that predicted job performance.

## Results

### Basic Descriptive Statistics

Tables 1, 2 list the mean score, standard deviation, and correlation between items and the total score for each item within the STEU-B and STEM-B scales, respectively. The mean scores on the STEU-B and STEM-B scales were 0.63 (*SD* = 0.19) and 0.60 (*SD* = 0.21), respectively. The Cronbach’s alpha coefficients for the STEU-B and STEM-B were 0.72 and 0.75, respectively. For the 19 STEU-B items, the correlations between items and the total score ranged from 0.33 to 0.54. For the 18 STEM-B items, the correlations between items and the total score ranged from 0.34 to 0.49. A significant gender difference was observed in the scores on the STEM-B (males: *M* = 0.58, *SD* = 0.22, *n* = 537; females: *M* = 0.63, *SD* = 0.18, *n* = 367; *t* = -3.15; *p* = 0.002; Cohen’s *d* = 0.26). However, no significant gender difference was observed in the scores on the STEU-B (males: *M* = 0.62, *SD* = 0.19, *n* = 537; females: *M* = 0.63, *SD* = 0.18, *n* = 367; *t* = -0.26; *p* > 0.05; Cohen’s *d* = 0.06).

**Table 1:** Descriptive statistics, item parameters and item information for STEU-B (*n* = 904).

Item	Descriptive statistics			Item parameters estimates			Information Total Test = 14.91	
	Mean	SD	r_it_	a_i_	b_i_	c_i_	Information	Information_max_
1	0.54	0.50	0.42^∗∗∗^	0.83	–0.18	0.01	0.78	0.19
2	0.30	0.46	0.40^∗∗∗^	0.91	0.97	0.05	0.68	0.15
3	0.47	0.50	0.42^∗∗∗^	0.91	0.28	0.03	0.78	0.18
4	0.66	0.47	0.54^∗∗∗^	1.81	–0.38	0.12	1.44	0.53
5	0.47	0.50	0.39^∗∗∗^	0.72	0.24	0.01	0.65	0.14
6	0.73	0.44	0.42^∗∗∗^	0.93	–1.22	0.01	0.87	0.22
7	0.75	0.44	0.46^∗∗∗^	1.32	–0.87	0.13	1.13	0.34
8	0.84	0.37	0.43^∗∗∗^	1.23	–1.67	0.01	1.20	0.39
9	0.73	0.44	0.43^∗∗∗^	0.98	–1.18	0.01	0.92	0.24
10	0.47	0.50	0.37^∗∗∗^	0.64	0.25	0.01	0.52	0.10
11	0.73	0.44	0.40^∗∗∗^	0.84	–1.38	0.01	0.78	0.19
12	0.47	0.50	0.37^∗∗∗^	0.83	0.63	0.13	0.53	0.11
13	0.73	0.47	0.40^∗∗∗^	0.82	–1.30	0.01	0.78	0.18
14	0.69	0.46	0.33^∗∗∗^	0.57	–1.48	0.01	0.43	0.09
15	0.66	0.47	0.42^∗∗∗^	0.83	–0.87	0.01	0.77	0.18
16	0.74	0.44	0.36^∗∗∗^	0.68	–1.64	0.01	0.60	0.13
17	0.77	0.42	0.39^∗∗∗^	0.80	–1.61	0.01	0.76	0.18
18	0.60	0.59	0.43^∗∗∗^	0.82	–0.54	0.01	0.75	0.17
19	0.57	0.49	0.37^∗∗∗^	0.67	–0.39	0.03	0.54	0.12

*STEU-B, brief version of Situational Test of Emotional Understanding; SD, standard deviation; r_*it*_, item-total correlation; a_*i*_, discrimination parameter; b_*i*_, difficulty parameter; c_*i*_, guessing parameter; Information_*max*_, maximum amount of item information. ^∗∗∗^*p* < 0.001.*

**Table 2:** Descriptive statistics, item parameters and item information for STEM-B (*n* = 904).

Item	Descriptive statistics			Item parameters estimates			Information Total Test = 16.27	
	Mean	SD	r_it_	a_i_	b_i_	c_i_	Information	Information_max_
1	0.47	0.50	0.43^∗∗∗^	0.89	0.22	0.01	0.79	0.19
2	0.54	0.50	0.43^∗∗∗^	0.83	–0.14	0.01	0.75	0.17
3	0.45	0.50	0.47^∗∗∗^	1.53	0.54	0.13	0.94	0.25
4	0.59	0.49	0.47^∗∗∗^	1.01	–0.39	0.01	0.97	0.26
5	0.67	0.47	0.43^∗∗∗^	0.87	–0.90	0.01	0.86	0.21
6	0.71	0.45	0.41^∗∗∗^	0.84	–1.16	0.01	0.82	0.20
7	0.62	0.49	0.42^∗∗∗^	0.82	–0.62	0.01	0.76	0.17
8	0.69	0.46	0.44^∗∗∗^	0.94	–0.91	0.01	0.94	0.24
9	0.73	0.44	0.46^∗∗∗^	1.09	–1.08	0.01	1.14	0.35
10	0.55	0.50	0.47^∗∗∗^	1.03	–0.17	0.01	0.96	0.26
11	0.64	0.48	0.49^∗∗∗^	1.16	–0.55	0.01	1.12	0.34
12	0.58	0.49	0.42^∗∗∗^	0.95	–0.07	0.12	0.77	0.18
13	0.48	0.50	0.49^∗∗∗^	1.62	0.39	0.12	1.01	0.28
14	0.63	0.48	0.46^∗∗∗^	0.96	–0.57	0.01	0.94	0.24
15	0.78	0.42	0.34^∗∗∗^	0.68	–2.00	0.01	0.61	0.13
16	0.36	0.48	0.41^∗∗∗^	1.31	1.00	0.12	0.71	0.16
17	0.50	0.50	0.40^∗∗∗^	0.79	0.08	0.01	0.69	0.15
18	0.83	0.38	0.46^∗∗∗^	1.23	–1.57	0.01	1.27	0.45

*STEM-B, brief version of Situational Test of Emotional Management; SD, standard deviation; r_*it*_, item-total correlation; a_*i*_, discrimination parameter; b_*i*_, difficulty parameter; c_*i*_, guessing parameter; Information_*max*_, maximum amount of item information. ^∗∗∗^*p* < 0.001.*

### Unidimensionality

In an IRT analysis, ensuring unidimensionality of the measurement is important. Therefore, CFA was conducted to test the unidimensionality of the STEU-B and STEM-B scales. The results showed that the one-factor model fitted the data on the Chinese version of the STEU-B well [*χ*^2^ = 232.80, *df* = 152, *GFI* = 0.97, *CFI* = 0.93, *IFI* = 0.93, RMSEA = 0.024, 90% *CI* = (0.018, 0.030)]. The fit indices for the STEM-B scale were similar [*χ*^2^ = 286.43, *df* = 135, *GFI* = 0.97, *CFI* = 0.90, *IFI* = 0.90, RMSEA = 0.035, 90% *CI* = (0.030, 0.041)]. These results provided supports for the unidimensionality of the STEU-B and STEM-B.

### Item Parameter Estimation and Information

The 3-PL model was used to fit the 19 STEU-B items. Table 1 shows the item parameters and the information for each item. The discrimination parameters ranged from 0.57 to 1.81, the difficulty parameters ranged from -1.67 to 0.97, and the guessing parameters ranged from 0.01 to 0.13. The item information for each item ranged from 0.43 to 1.44, and the maximum amount of item information ranged from 0.09 to 0.53. The total test information for the STEU-B scale was 14.91, and the point of maximum test information on the 𝜃 scale was -0.61, which suggested that the STEU-B scale can provide more sufficient information for individuals with low emotional understanding ability than those with high emotional understanding ability.

The 3-PL model was used to fit the 18 STEM-B items. Table 2 shows the item parameters and the information for each item. The discrimination parameters ranged from 0.68 to 1.62, the difficulty parameters ranged from -2.00 to 1.00, and the guessing parameters ranged from 0.01 to 0.13. The item information for each item ranged from 0.61 to 1.27, and the maximum amount of item information ranged from 0.13 to 0.45. The test information for the STEM-B scale was 16.27, and the point of maximum test information on the 𝜃 scale was -0.42, which suggested that the STEM-B scale can provide more sufficient information for individuals with low emotional management ability than those with high emotional management ability.

### Correlations of STEU-B, STEM-B and Criterion Variables

The partial correlations among the STEU-B score, the STEM-B score, and other criterion-related variables by controlling age, gender, and job tenure are shown in Table 3. The STEU-B score was significantly correlated with the STEM-B score (*r* = 0.32, *p* < 0.001). The STEU-B score was significantly and negatively correlated with psychological strain, LAUA and overall negative affect at work. It significantly and positively correlated with LAPA, overall positive affect, the anxiety-comfort score, and the depression-enthusiasm score, job satisfaction, and supervisor-rated general job performance. The STEM-B score was significantly associated with all measured criterion-related variables in the expected directions.

**Table 3:** Descriptive statistics and partial correlations of all variables.

	Mean	SD	1	2	3	4	5	6	7	8	9	10	11	12
1. STEU-B	0.63	0.19	*0.72*											
2. STEM-B	0.60	0.21	0.32***	*0.75*										
3. Psychological strain	2.57	0.66	–0.19***	–0.22***	*0.87*									
4. HAPA	4.17	1.02	0.06	0.25***	–0.46***	*0.87*								
5. LAPA	4.17	1.04	0.12***	0.23***	–0.46***	0.60***	*0.85*							
6. HAUA	2.90	0.81	–0.06	–0.20***	0.50***	–0.30***	–0.36***	*0.82*						
7. LAUA	2.34	0.73	–0.19***	–0.24***	0.59***	–0.32***	–0.29***	0.61***	*0.83*					
8. Positive affect	4.17	0.92	0.10**	0.27***	–0.51***	0.89***	0.89***	–0.37***	–0.34***	*0.90*				
9. Negative affect	2.62	0.69	–0.14***	–0.25***	0.60***	–0.35***	–0.36***	0.91***	0.88***	–0.40***	*0.88*			
10. AC	4.13	0.76	0.11**	0.27***	–0.57***	0.57***	0.87***	–0.77***	–0.52***	0.81***	–0.73	*0.84*		
11. DE	4,41	0.72	0.14***	0.31***	–0.62***	0.88***	0.58***	–0.53***	–0.74***	0.81***	–0.70***	0.67***	*0.84*	
12. Job satisfaction	3.62	0.63	0.22***	0.30***	–0.37***	0.38***	0.25***	–0.22***	–0.34***	–0.31***	0.29***	0.44***	–0.31***	*0.81*
13. Job performance	3.05	0.67	0.11**	0.21***	–0.31***	0.20***	0.24***	–0.22***	–0.20***	–0.24***	0.28***	0.25***	–0.24***	0.17***

*STEU-B, brief version of Situational Test of Emotional Understanding; STEM-B, brief version of Situational Test of Emotional Management; HAPA, high-activation pleasant affect; LAPA, low-activation pleasant affect; HAUA, high-activation unpleasant affect; LAUA, low-activation unpleasant affect; AC, anxiety-comfort; DE, depression-enthusiasm. Diagonal italic numbers represent the internal consistency coefficient of the scale. *n* = 632 for correlations with job performance, *n* = 904 for other correlations. ^∗∗^*p* < 0.01, ^∗∗∗^*p* < 0.001.*

### Regression Analysis Predicting Job Performance

To further explore the differential predictive power of STEU-B and STEM-B on job performance, a hierarchical regression analysis predicting job performance was conducted. Independent variables and outcome variable were standardized to control the size of the effects. First, gender, age, and job tenure were entered as control variables. Second, the STEU-B score was entered into the regression. The results showed that this score significantly predicted job performance (β = 0.11, *p* = 0.008). Third, the STEM-B score was entered. The results showed that the STEM-B score significantly predicted job performance (β = 0.20, *p* < 0.001), whereas the coefficient of the STEU-B score became insignificant (β = 0.04, *p* > 0.05). Moreover, bootstrap results suggested that the standardized coefficient for the indirect effect of the STEU-B score on job performance through the STEM-B score was significant [effect = 0.07; 95% *CI* = (0.37, 0.11)].

## Discussion

This study examined the psychometric properties of STEU-B and STEM-B using the IRT method and their criterion validity in a sample of 904 Chinese employees. The internal consistencies of the Chinese versions of the STEU-B and STEM-B scales were found to be adequate; both were above 0.70. The mean scores on STEU-B and STEM-B in the Chinese context were close to those on the original version in the Western context (Allen et al., 2014, 2015). Previous studies reported that east Asians performed worse on MSCEIT than did North Americans (Mayer et al., 2002). This cultural difference in the scores on the performance-based EI test was in part because the test was developed in the west, and the correct answers to problems about emotions in the test varied across different cultures (Moon, 2011; Côté, 2014). However, our results indicated that the correct answers and scoring systems of STEU-B and STEM-B that were developed in the west were also applicable in the Chinese context.

Furthermore, the IRT analyses revealed that all of the items within the original STEU-B and STEM-B scales had good discrimination parameters in the Chinese context (moderate to high level). Moreover, the difficulty values of these items were evenly spaced, ranging from -2.00 to 1.00. The item information for each item was then computed as a function of item parameters. The maximum amount of item information ranged from 0.09 to 0.53 in this study, which exceeded the cutoff value of 0.05 suggested by Allen et al. (2014). These results were in line with previous findings, which showed that the items included in the STEU-B and STEM-B were able to distinguish different levels of EI effectively and provide sufficient item information (Allen et al., 2014, 2015). The inspection of both the IIFs and TIFs revealed that the Chinese versions of STEU-B and STEM-B had uneven information functions, and that STEU-B and STEM-B provided the maximum information for individuals with a trait value of -0.61 and a trait value of -0.42, respectively. Thus, similar to the English version, the Chinese versions of STEU-B and STEM-B were proved to be more useful in identifying individuals with poor to average emotional understanding and emotional management (Allen et al., 2014, 2015). Taken together, these results indicated that the psychometric properties of the Chinese versions of STEU-B and STEM-B were satisfactory, and that the original scoring systems of these scales were applicable in the Chinese context.

The criterion validity of the Chinese versions of STEU-B and STEM-B was evaluated by determining whether the STEU-B and STEM-B scores were related to several work criteria in meaningful ways. Consistent with substantial EI research reported in the west, which suggested that EI played an important role in stress management and job satisfaction (Oginska-Bulik, 2005; Gallagher and Vella-Brodrick, 2008; Vratskikh et al., 2016), the Chinese versions of the STEU-B and STEM-B scores were significantly related to a reduction in employees’ psychological strain and an increase in their job satisfaction. The results also demonstrated that the STEM-B score had positive relationships with both the HAPA and LAPA, and negative relationships with both the HAUA and LAUA at work, whereas the STEU-B score was only weakly associated with LAPA (e.g., being at ease) and LAUA (e.g., feeling dejected). These results were in line with previous studies that suggested that regulation of emotion was a more predictive EI dimension of work-related effects than of emotional understanding (Kafetsios and Zampetakis, 2008; Parke et al., 2015). Although the relationships between the STEU-B score and work-related effects were not expected, these results indicated that the employees with a high degree of emotional understanding experienced lower levels of LAUA in the Chinese organizational context. Both STEU-B and STEM-B were also significantly associated with double-quadrant dimensions affective scores. However, the correlations between STEU-B and these scores were very weak. Overall, the observed correlations between STEU-B and criteria indicated that the STEU-B had a stronger correlation with job satisfaction which involved the cognitive evaluation regarding different aspects of work, whereas the associations between STEU-B and affect-related scores were weaker. These results were consistent with the theoretical argument that emotional understanding was the most “cognitive” EI branch, which had a strong association with abstract reasoning and emotional information-processing (Mayer et al., 2001).

Our results also demonstrated that both the STEU-B and STEM-B scores were related to the supervisor-rated general job performance, and the association between STEM-B score and job performance was stronger than that between STEU-B score and job performance. The correlations in this study were similar to those reported in previous meta-analyses (Joseph and Newman, 2010; O’Boyle et al., 2011). The regulating emotions branch is the highest and most complex EI branch, which involves motivational, emotional, and cognitive factors. Thus, it may facilitate employees’ general job performance by achieving more adaptive mood states, obtaining valuable resources, forming better relationships with coworkers or customers, and promoting personal growth. Furthermore, in line with the cascading model of EI, which proposed that the higher branches of abilities (e.g., emotional regulation) were developed on the basis of the lower branches of abilities (e.g., emotional understanding) (Joseph and Newman, 2010; Newman et al., 2010), our results indicated that the understanding of emotions in specific situations may impact the management of emotions, such as the strategies we use to regulate our emotions, which in turn contribute to job performance. The practical implication of this is that it is meaningful to utilize some training programs to improve the emotional understanding of ability EI before emotional regulation to enhance employee’s job performance.

The STEU-B and STEM-B target the two higher, strategic branches of the ability EI that are important in the organizational context. The STEU-B and STEM-B are theoretically based and provide sufficient test information with fewer items, which is time-saving. Therefore, it would be very useful when testing time is severely limited and for researches that focus on strategic EI rather than experiential EI. Moreover, unlike MSCEIT which is a commercial test with scoring performed by a test company, the items selection and scoring systems of STEU-B and STEM-B are provided clearly to EI researchers. Thus, it is possible to further develop and improve these instruments. However, there are some limitations to this method of measurement. The item selection was based on test information curves, and this might have decreased the measurement precision for respondents whose ability lay outside of the mean (Allen et al., 2015). The mean scores of the Chinese versions of STEU-B and STEM-B in the current study were also found to be higher than those of the original full-length versions (MacCann and Roberts, 2008), indicating that the easier items were selected. Thus, the STEU-B and STEM-B would be more useful in populations where lower levels of emotional understanding and management are assumed.

Some limitations of this study and directions for further research should be addressed. First, the sample of this study was derived from a high-tech organization in three major cities of China, where the level of educational attainment was relatively high. In addition, these participants were relatively young, and different patterns in EI may be affected by individuals’ growth. Therefore, future studies should include broader samples of different occupations, education levels, socioeconomic backgrounds, and age groups to generalize these measurements. Second, although we provided evidence for the criterion validity of STEU-B and STEM-B in a Chinese organizational setting by examining their relationships with several important work-related criteria, the incremental validity was not examined since we did not control other individual different variables that predicted work-related criteria, such as cognitive ability, personality traits, and self-reported EI (Joseph and Newman, 2010; O’Boyle et al., 2011). Recent meta-analysis studies provided support for the incremental validity of EI in predicting work attitude (Miao et al., 2017) and job performance (Miao et al., 2018) while controlling for the big five personality traits and cognitive ability. Therefore, it is of great importance to explore the incremental validity of STEU-B and STEM-B in the organizational context by including these variables. Third, the associations between ability EI and general job performance were proved to be weak in our study. It has been proposed that the relationships between EI and work outcomes depend on the job or employment setting. Thus, considering other variables that is related to the specific work situation, or other work criteria are also important. For example, further studies can relate the STEU-B and STEM-B to other work criteria, such as emotional labor, contextual performance, and leadership. Fourth, the underlying cognitive processes may be different for different format (multiple-choice or rate-the-extent) (MacCann and Roberts, 2008), thus future research could explore the influence of thinking mode in EI research. Finally, we did not consider the influence of cultural values on EI and work-related outcomes, such as collectivism and long-term orientation (Miao et al., 2018). Researchers should incorporate these factors when delving into this topic in the future.

## Conclusion

This study examined the applicability of two performance-based EI tests, namely STEU-B and STEM-B, in a sample of 904 Chinese employees. The internal consistencies were acceptable. The item parameters provided by the IRT analyses showed good discriminatory power and reasonable variation in difficulty across all the items within the STEU-B and STEM-B scales. Moreover, the scores on STEU-B and STEM-B were associated with several emotion- and work-related criteria in meaningful ways. Taken together, the Chinese versions of STEU-B and STEM-B scales were found to be psychometrically adequate measurements which might be useful to capture employees’ emotional understanding and emotional regulation as alternative ability EI tests. Further research should focus on further validation in broader work contexts, and in relation with various personality traits, intelligence, and work-related outcomes.

## Ethics Statement

All procedures performed in this study were in accordance with the ethical standards of the institutional research committee and with the 1964 Helsinki Declaration and its later amendments or comparable ethical standards.

## Author Contributions

SY collected the data, analyzed and interpreted the data, wrote the manuscript, and was involved in the study conception and design. YF collected the data, analyzed and interpreted the data, and was involved in manuscript preparation and revision. YX conceived and designed the study, analyzed and interpreted the data, reviewed and edited the manuscript, and provided final approval of the version. YL conceived and designed the study, analyzed and interpreted the data, and was involved in manuscript preparation.

## Conflict of Interest Statement

The authors declare that the research was conducted in the absence of any commercial or financial relationships that could be construed as a potential conflict of interest.
